# Health-related quality of life as a predictor of pediatric healthcare costs: A two-year prospective cohort analysis

**DOI:** 10.1186/1477-7525-2-48

**Published:** 2004-09-10

**Authors:** Michael Seid, James W Varni, Darron Segall, Paul S Kurtin

**Affiliations:** 1 RAND Health, 1700 Main Street, M-28, Santa Monica, California, 90407, USA; 2 Department of Landscape Architecture and Urban Planning, College of Architecture Texas A&M University, 3137 TAMU, College Station, Texas 77843, USA; 3 Department of Pediatrics, College of Medicine, Texas A&M University, 3137 TAMU, College Station, Texas 77843, USA; 4 MHS., San Diego, California, USA; 5 Center for Child Health Outcomes, 3020 Children's Way, San Diego, CA, 92123, USA

**Keywords:** Health-related quality of life, PedsQL™, prediction, healthcare costs, managed care.

## Abstract

**Background:**

The objective of this study was to test the primary hypothesis that parent proxy-report of pediatric health-related quality of life (HRQL) would prospectively predict pediatric healthcare costs over a two-year period. The exploratory hypothesis tested anticipated that a relatively small group of children would account for a disproportionately large percent of healthcare costs.

**Methods:**

317 children (157 girls) ages 2 to 18 years, members of a managed care health plan with prospective payment participated in a two-year prospective longitudinal study. At Time 1, parents reported child HRQL using the Pediatric Quality of Life Inventory™ (PedsQL™ 4.0) Generic Core Scales, and chronic health condition status. Costs, based on health plan utilization claims and encounters, were derived for 6, 12, and 24 months.

**Results:**

In multiple linear regression equations, Time 1 parent proxy-reported HRQL prospectively accounted for significant variance in healthcare costs at 6, 12, and 24 months. Adjusted regression models that included both HRQL scores and chronic health condition status accounted for 10.1%, 14.4%, and 21.2% of the variance in healthcare costs at 6, 12, and 24 months. Parent proxy-reported HRQL and chronic health condition status together defined a 'high risk' group, constituting 8.7% of the sample and accounting for 37.4%, 59.2%, and 62% of healthcare costs at 6, 12, and 24 months. The high risk group's per member per month healthcare costs were, on average, 12 times that of other enrollees' at 24 months.

**Conclusions:**

While these findings should be further tested in a larger sample, our data suggest that parent proxy-reported HRQL can be used to prospectively predict healthcare costs. When combined with chronic health condition status, parent proxy-reported HRQL can identify an at risk group of children as candidates for proactive care coordination.

## Background

Predicting healthcare costs for pediatric populations has been challenging [1]. Although population-based risk prediction and case-mix adjustment can be used to inform policy, set rates, and compare outcomes across providers [2], a more immediate concern for healthcare providers is to clinically manage their enrolled population. In a prospective payment system with predetermined funding limits, providers must be able to proactively case-manage those enrollees at greatest risk of poor health while remaining within designated budget constraints. If healthcare providers knew in advance – for example at the time of health plan enrollment – which children were at the greatest risk for future health problems, then healthcare resources could be proactively targeted to those children in order to minimize or prevent morbidity and associated healthcare costs.

Researchers working with adult populations have linked health status with several important outcomes. In general populations, self-reported health status has been shown to be a predictor of future health services charges [3], the use of physician services and mortality in working-age adults [4], and of frailty in the elderly [5]. For chronically ill adults, self-rated health status is an independent predictor of physiologic health in diabetes and hypertension[6], and self-reported quality of life is an independent predictor of survival in cancer patients [7]. For the hospitalized elderly, functional status [8-10] and depressive symptoms [11] have been shown to be predictive of resource utilization and mortality. Several researchers have demonstrated that both diagnostic information and self-rated health status are associated with costs for general adult populations [12,13]. In pediatric populations, diagnosis-based classification systems have achieved some degree of association with healthcare costs [14,15]. However, there remain limitations with current pediatric healthcare cost prediction methods, including the underestimation of healthcare costs for chronically ill children [14]. The ideal pediatric cost prediction model for clinical management would predict healthcare costs proactively in those patients at greatest risk.

Increasingly, health-related quality of life (HRQL) has become recognized as an important health outcome, some contend *the *most important outcome in child health services research [16-18]. Researchers have made great strides in conceptualizing and measuring HRQL for children [19-27]. HRQL has been shown to be responsive to treatment in children with rheumatic disease [28], and to be related to treatment status in children with cancer [20,29], to chronic health condition status in a general sample [19], to severity of illness within children with cardiac diagnoses [30], and to parent reports of primary care quality [31] and barriers to care [32]. Measuring HRQL in large populations has several distinct benefits. It can aid in identifying subgroups of children who are at-risk for health problems [33], in determining the burden of a particular disease or disability [34], and, at least in general populations, in informing efforts aimed at prevention and intervention [13]. While self-report is considered the standard for measuring perceived HRQL as an outcome, it is typically parents' perceptions of their children's HRQL that influence healthcare utilization [35,36].

Consequently, the objective of this study was to test the primary hypothesis that parent proxy-report of pediatric HRQL would prospectively predict pediatric healthcare costs over a two-year period. The exploratory hypothesis tested anticipated that a relatively small group of children would account for a disproportionately large percent of healthcare costs.

## Method

### Participants and Settings

The study took place in San Diego, California between January 1998 and December 2000. We recruited members of a 50,000-member federally supported (Medicaid) managed care health plan. Additional inclusion criteria were that children be between 2 and 18 years of age and that the parent be able to speak either English or Spanish. We exclude children under 2 years of age because the PedsQL™ does not assess parent proxy-report HRQOL below age 2 years. In order to maximize the heterogeneity of the sample, subjects were recruited from three types of healthcare settings: children presenting at pediatricians' offices for scheduled well-child checks (n = 18, 5.7%), children at one of two hospital specialty clinics – orthopedics (n = 6, 1.9%) and cardiology (n = 7, 2.1%) – or children who had been seen at the hospital or its outpatient clinics at least three months previously (n = 286, 90.3%). The data reported here were collected as part of the initial field test to assess the reliability and validity of the PedsQL™ 4.0 Generic Core Scales [19]. Only pediatric patients reported being members of the federally supported managed care health plan are included in the current data analysis.

## Measures

### The PedsQL™ 4.0 (Pediatric Quality of Life Inventory™ 4.0) Generic Core Scales

The PedsQL™ 4.0 Generic Core Scales [19] were designed to measure the core physical, mental and social health dimensions as delineated by the World Health Organization [37], and to additionally include role (school) functioning. The 23-item PedsQL™ 4.0 encompasses both physical functioning (8 items) and psychosocial (emotional, social, role) functioning (15 items) and is comprised of parallel child self-report and parent proxy-report formats. The parent proxy-report form is designed to assess the parent's perceptions of their child's HRQL. Parent proxy-report includes ages 2–4 (toddler), 5–7 (young child), 8–12 (child), and 13–18 (adolescent).

Higher PedsQL™ 4.0 scores indicate better HRQL. To create Scale Scores, the mean is computed as the sum of the items divided by the number of items answered (this accounts for missing data). If more than 50% of the items in the scale are missing, the Scale Score is not computed. Imputing the mean of the completed items in a scale when 50% or more are completed is generally the most unbiased and precise method [38]. Because parent proxy-report of HRQL has been shown to be related to utilization [35,36], we used only the parent proxy-report Physical Functioning and Psychosocial Functioning Summary Scales of the PedsQL™ 4.0 in the current investigation.

### Chronic health condition status

Parents were asked to report on the presence of a chronic health condition for their child. They read the following statement: "A chronic health condition is: (1) a physical or mental health condition (2) that has lasted or is expected to last at least 6 months and (3) interferes with your child's activities." They then responded with yes or no to the question "In the past 6 months, has your child had a chronic health condition?" If yes, the parents were asked to identify the name of the chronic health condition. Parents who answered yes or who gave the name of a chronic health condition were coded as having a child with a chronic health condition. This method has been used in previous work [19,31], and the PedsQL™ 4.0 scores for the two groups defined using this method (with and without chronic health condition) are very similar to those observed in other studies [33].

### Healthcare Costs

Healthcare costs were calculated as the dollar amount paid by the health plan per patient. We first determined patients' eligibility from the health plan's eligibility data files for three consecutive cumulative periods: 0–6 months, 0–12 months, and 0–24 months after the date they completed the PedsQL™ 4.0. A pediatric patient was considered eligible for health plan benefits for those periods if they were eligible for at least 5 months out of the 6-month period. We then electronically captured healthcare costs (the dollar amount paid by the health plan) for each pediatric patient for those periods in which they were eligible. We did this by matching each eligible pediatric patient with the health plan's existing databse of claims and encounter data. These data include the dollar amount spent by the health plan. Healthcare costs included hospital and emergency room costs, professional fees, durable medical equipment, home health, specialty clinic, and primary care costs. We did not have access to pharmacy or mental health costs.

In California, the site of the study, treatment for 22 specific diagnoses is "carved out," or paid through a separate program (called California Children's Services; CCS) regardless of a child's health plan membership. Thus, for health plans in California, treatment of CCS-covered diagnoses might not be measured in calculating utilization. However, because California's carve out may differ from other states' methods of financing treatment for these diagnoses, and to more completely describe healthcare costs, we included the costs for procedures covered by CCS in our healthcare costs calculations. To derive these costs, we linked the procedure codes on the health plan's CCS referral with the federally supported health plan's fee schedule. These data thus represent the dollar amount the health plan would have spent had the services not been carved out.

### Procedure

A convenience sample – subects were recruited nonsystematically when research assistants were available – was recruited at pediatrician offices and specialty clinics. These pediatric patients were identified through examination of the clinic appointment schedules. At these sites, parents of children identified as possible study participants were informed of the study by one of the research assistants after checking in for their appointment, but before being seen by their healthcare provider. Written informed consent included permission for the researchers to examine the medical record to assess utilization. After written informed consent was obtained, the parent completed the proxy-report version of the PedsQL™ 4.0. The research assistant was available at all times to answer any questions.

A random sample was recruited from children and adolescents ages 2–18 years who had been seen as inpatients or outpatients at Children's Hospital and Health Center between April 1 and June 30, 1998, and who were members of the health plan. This sample excluded children with a discharge status of expired, children whose payer was from the victim/witness fund, and children whose parents had requested their phone number and address to be kept private. Research assistants called parents of children on this list and obtained verbal informed consent. The research assistant verbally administered the PedsQL™ 4.0 to parents. This research protocol was approved by the institutional review board at Children's Hospital and Health Center, San Diego (#98-020).

### Statistical analysis

We pooled the data from the two samples. Previous reasearch on the PedsQL™ has documented the lack of mode of administration effects [19,20]. In order to test the primary hypothesis that HRQL would prospectively predict healthcare costs, multiple linear regression analyses were conducted. We examined the association between age, gender, chronic health condition status (variables typically used by health plans to predict risk), and PedsQL™ 4.0 scores with healthcare costs at each of the three cumulative follow-up periods. We did not use socioeconomic status, as eligibility criteria for membership in the health plan requires families to have incomes below a certain level, and this restricts the range of this variable. Four models were constructed for each follow-up period. Model 1 included age and gender only, Model 2 included age, gender, and chronic health condition status, Model 3 included age, gender, and PedsQL™ 4.0 scores, and Model 4 included age, gender, chronic health condition status, and PedsQL™ 4.0 scores. We report the adjusted *R*^2^, a measure of the percent of variance in the dependent variable accounted for by the predictor variables while adjusting for the complexity of the model, and the standardized regression coefficient, or beta, for each predictor.

PedsQL™ 4.0 scores were skewed toward the high end of the scale and were transformed by taking the square root of the reverse of the score (sqrt(100-score)) in order to create a more normal distribution. The distribution of cost data was skewed to the lower end, with many children having little cost and a relatively smaller number of children having high costs. These data were normalized by taking the log of the costs.

In order to explore whether HRQL and chronic health condition status together would define a relatively small subset of enrollees who accounted for a disproportionately large percent of healthcare costs, we divided the sample into quintiles based on the PedsQL™ 4.0 Physical Functioning Scale score and into two groups based on chronic health condition status. Those children who fell in the lowest PedsQL™ 4.0 quintile and who reported the presence of a chronic health condition were assigned to the high-risk group. We describe the percent of costs, per member costs, and per member per month costs per child accounted for by this high risk group.

## Results

### Descriptive Statistics

Data was collected from the parents of 317 children (157 girls, 160 boys) ages 2 to 18 years. The average age of the children was 8.3 years (SD = 4.14) with a range of 2.03 to 17.13 years. The sample was heterogeneous with respect to race/ethnicity, with 76 (25.4%) White non-Hispanic, 155 (51.8%) Hispanic, 39 (13.0%) Black non-Hispanic, 6 (2.0%) Asian/Pacific Islander, 3 (1.0%) American Indian or Alaskan Native, 20 (6.7%) Other, and 19 (6.0%) missing. With respect to mother's education, 36.4% had less than a high school education, 46.6% had a high school diploma or some college, and 7.0% were college graduates or beyond (18.8% missing). The measures were administered in two languages – English (n = 233, 73.6%) and Spanish (n = 84, 26.4%). The sample represented both chronically ill (n = 102, 32.1%) and healthy children (n = 215, 67.9%), based on parent report of the presence of a chronic health condition. Table 1 presents the chronic health conditions reported by parents for the high risk group and the non-high risk group.

**Table 1:** Parent-reported chronic health conditions, by high risk group status.

		High risk group?		Total
		no	yes	

name of condition	None	223	0	223
	ADHD	7	1	8
	allergies	1	0	1
	arthritis	2	0	2
	asthma	25	8	33
	autism	2	1	3
	back and leg pain	0	1	1
	Battan disease	0	1	1
	bi-polar	1	0	1
	breathing problems	1	0	1
	bronchitis	2	0	2
	cancer	1	0	1
	cerebral palsy	2	2	4
	chronic ear infections	2	0	2
	cough	2	0	2
	CSF leak	0	1	1
	depression	1	0	1
	developmental delay	1	0	1
	dislocated shoulder	0	1	1
	doesn't produce salt	1	0	1
	Down Syndrome	1	0	1
	ear problems	0	1	1
	epilepsy	1	0	1
	gallbladder problems	1	0	1
	gastrointestinal problem	1	0	1
	hearing	1	0	1
	heart murmur	2	0	2
	heart problem	1	1	2
	kidney reflux	1	0	1
	leukemia	1	0	1
	May Hegglin syndorme	1	0	1
	migraine headaches	0	1	1
	muscle condition	0	1	1
	muscular dystrophy	0	1	1
	osteomyelitis	1	0	1
	pierre robyn syndrome	1	0	1
	reactive airway disease	1	0	1
	Rett syndrome	1	0	1
	scleroderma	0	1	1
	scoliosis	0	1	1
	seizures	3	1	4
	speech	1	0	1
	spina bifida	0	2	2
	stomach aches	1	0	1
	stomach problems	1	0	1
	unknown genetic syndrome	0	1	1
	urinary tract infections	1	0	1

There were no differences found in PedsQL™ scores between the group sampled at well-child checks or specialty clinics and that sampled by phone.

All 317 children were enrolled in the health plan after 6 months, with 314 (99.0%) enrolled after 12 months, and 244 (76.9%) after 24 months. There were no differences between those enrolled versus not enrolled at 24 months in percent with a chronic health condition, race/ethnicity, mother's education, or PedsQL™ scores. The cost per member per month (pmpm) for this sample, which represents the total cost divided by the number of members divided by the number of months enrolled, was $149 at 6 months, $137 at 12 months, and $115 at 24 months.

The sample included 4,954 claims (there are multiple claims in a single clinical encounter) over the 24 months. The largest category of visits was for upper respiratory infections (URIs) and related infections (10.96%). Asthma, other infections, otitis media, and pain each account for 5 to 6% of visits, with acute orthopedic conditions accounting for 2.6% of visits. These most common diagnoses account for more than a third (38.7%) of the visits, the rest is comprised of a large number of relatively low-frequency diagnoses. This distribution of diagnoses is similar to the epidemiology of childhood illness, in that much of pediatric morbidity is accounted for by a large number of relatively low frequency diagnoses [39,40].

Table 2 displays the descriptive statistics for the PedsQL™ 4.0 parent proxy-report at Time 1. Consistent with previous PedsQL™ 4.0 findings, [19] chronically ill children had lower HRQL scores than healthy children (Table 2).

**Table 2:** Descriptive Statistics for PedsQL™ 4.0 scores

	N	Mean	SD	Minimum	Maximum	*t*^	*df*	*p*
Total Sample								
Total Scale Score	316	84.38	13.67	25.00	100.00			
Physical Functioning	316	85.39	19.87	0.00	100.00			
Psychosocial Functioning	317	83.78	13.73	32.14	100.00			
Children with Chronic Health Condition								
Total Scale Score	101	79.26	16.66	25.00	100.00	-4.73	313	0.001
Physical Functioning	100	79.69	25.00	0.00	100.00	-3.59	313	0.001
Psychosocial Functioning	102	79.27	16.59	32.14	100.00	-4.12	314	0.001
Children without Chronic Health Condition								
Total Scale Score	214	86.82	11.29	44.44	100.00			
Physical Functioning	215	88.17	16.30	10.00	100.00			
Psychosocial Functioning	214	85.91	11.61	48.33	100.00			

^comparing chronically ill to healthy children

### Multiple regression analysis

Table 3 displays the results of the multiple regression analyses predicting healthcare costs for 6, 12, and 24 month follow-up. As can be seen, Model 1, with age and gender as the only predictors variables, did not account for significant variance in costs. Model 2 shows that age and gender, with chronic health condition status accounted for an increasing percentage of costs as the follow-up time lengthened. This pattern holds true as well for Model 3, which included age, gender, and the PedsQL™ 4.0 scores. Model 4, comprised of age, gender, chronic health condition status, and PedsQL™ 4.0 scores, accounted for the most variance, explaining 10.1% 14.4% and 21.2% of the variance in healthcare costs at 6, 12, and 24 month follow-up intervals. Inspection of the standardized regression coefficients for each predictor in Model 4 shows that, of the four predictors used, chronic health condition status and the PedsQL™ 4.0 Physical Functioning Scale scores consistently accounted for the greatest amount of variance.

**Table 3:** Adjusted R-square (in bold) and standardized regression coefficients (betas) for models predicting costs at 6, 12, and 24 months

	Follow up		
	6 Months (N = 318)	12 Months (N = 315)	24 Months (N = 245)
Model 1 Adjusted R-square	**0.010**	**0.001**	**0.004**
Beta			
Age	-0.067	-0.037	-0.030
Gender	.11	0.063	0.108
Model 2 Adjusted R-square	0.049**	0.066***	0.130***
Beta			
Age	-0.063	-0.031	0.008
Gender	0.118*	0.076	0.115
Chronic Health Condition	0.180**	0.265***	0.360***
Model 3 Adjusted R-square	0.089***	0.103***	0.122***
Beta			
Age	-0.103	-0.091	-0.079
Gender	0.080	0.030	0.076
PedsQL Physical Functioning	0.291***	0.353***	0.379***
PedsQL Psychosocial Functioning	0.004	0.057	0.081
Model 4 Adjusted R-square	0.101***	0.144***	0.212***
Beta			
Age	0.098	-0.083	-0.043
Gender	0.086	0.040	0.080
Chronic Health Condition	0.126^+^	0.214***	0.312***
PedsQL Physical Functioning	0.275***	0.326***	0.340***
PedsQL Psychosocial Functioning	0.025	0.093	0.123^+^

^+ ^= *p *< 0.05; * = *p *< 0.01; *** = *p *< 0.001; *** = *p *< 0.0001; R^2 ^= percent variance accounted for.

### Defining the high risk group

We used the two variables accounting for most of the variance in the regression analysis – the PedsQL™ 4.0 Physical Functioning scores and chronic health condition status – to describe the percentage of costs accounted for by different groups of children. In order to create a single denominator for the percentages, we used the 241 children continuously enrolled in the health plan with complete data for this set of analyses. To create quintiles, we determined the values that divided the sample into five equal-sized groups based on PedsQL™ 4.0 Physical Functioning Scale scores. Enrollees with a score of less than 75 on the PedsQL™ 4.0's 0–100 scale were in the first quintile (N = 51; 21.0%). The second quintile (N = 45; 18.5%) was bounded by the scores 75.0 to 90.624, the third quintile (N = 48; 19.6%) by the scores 90.625 to 96.874, the fourth quintile by the scores 96.875 to 100 (N = 18; 7.3%), and the fifth quintile consisted of enrollees scoring 100 (N = 81; 33.4%). Because the distribution of these PedsQL™ 4.0 scores was skewed, we combined the fourth and fifth quintiles (N = 99; 40.7%; 2 missing).

Table 4 shows the percentage of total costs accounted for by children across PedsQL™ 4.0 Physical Functioning Scale quintiles and chronic health condition status, for the three cumulative follow up periods. As can be seen, children in the high risk group (the subset of chronically ill children in the lowest quintile), account for a disproportionately large share of healthcare costs. This group, comprising just 8.7% of the sample, accounted for 37.42% of the healthcare costs over six months, 59.16 % of costs over 12 months, and 61.74% of costs over 24 months.

**Table 4:** Percent of cost at each follow up period, by PedsQL quintile and chronic health condition status (n = 241)

	PedsQL Physical Functioning Quintile							
	Lowest quintile (N = 51)		2nd quintile (N = 45)		3rd quintile (N = 48)		4th and 5th quintiles (N = 99)	
	Chronic Health Condition		Chronic Health Condition		Chronic Health Condition		Chronic Health Condition	
	Yes (N = 21)	No (N = 30)	Yes (N = 18)	No (N = 27)	Yes (N = 12)	No (N = 36)	Yes (n = 26)	No ( = 73)

% of cost, 6 months	**37.43**	9.35	26.76	9.30	0.33	6.95	7.59	2.30
% of cost, 12 months	**59.16**	4.70	20.56	4.54	0.30	3.63	5.25	1.85
% of cost, 24 months	**61.74**	3.38	22.06	2.86	0.41	2.64	4.87	2.03

**Bold **= High-risk group

Table 5 shows the total costs, the per member costs, and the per member per month (pmpm) costs for the high risk group and the not high risk group over the three follow-up periods. As can be seen, the high risk group was an extremely costly subset of enrollees for each of the cumulative 6 month periods, as measured by total, per member, or pmpm costs. Pmpm costs were quite disparate between the high risk group and other enrollees. For the high risk group at 6 months, pmpm was $432 (vs. $66 for the other patients), at 12 months pmpm was $809 (vs. $61), and at 24 months, pmpm was $722 (vs. $60).

**Table 5:** Total costs, per member costs, and per member per month costs for high-risk* (N = 21) and not high-risk (N = 231) enrollees.

	**Total**		**Per member**		**Per member per month**	
Time Period	High-risk	Not high-risk	High-risk	Not high-risk	High-risk	Not high-risk
	
6 months	$54,493	$91,484	$2,595	$396	$432	$66
12 months	$203,875	$168,022	$9,708	$727	$809	$61
24 months	$363,822	$330,846	$17,325	$1,432	$722	$60

*High risk is defined as parent reported chronic health condition and scoring in lowest quintile on PedsQL™ 4.0

## Discussion

This study tested the primary hypothesis that HRQL could prospectively predict healthcare cost in pediatric patients in a managed care environment. We measured age, chronic health condition status, and PedsQL™ 4.0 scores at Time 1, and prospectively measured utilization, via costs based on claims and encounter data, for three cumulative periods. These data demonstrate that parent-reported HRQL, as measured by the PedsQL™ 4.0, and chronic health condition status each accounted for significant variance in healthcare costs over 6, 12, and 24 months. The data further show how these two predictor variables, chronic health condition status, and PedsQL™ 4.0 Physical Functioning scores, define a relatively small group of enrollees that accounted for a large percentage of total healthcare costs.

This high risk group displays disproportionately high costs as early as 6 months, and their pmpm costs peak at one year. This suggests the importance of managing high risk enrollees as soon as they are identified, perhaps as early as their initial enrollment. It also implies the potential for significant return on investment for better case management, even in the first six months of enrollment. The high risk group's costs remain disproportionately high throughout the 24 months of the study. This fact suggests that the method used here for identifying the high risk group succeeded in identifying children with high ongoing care needs and costs, as opposed to children with one-time health care needs. An anomalous finding was that children in the third quintile on PedsQL™ scores who had chronic health conditions were, for an unexplained reason, much less costly than their peers.

It is worth comparing the mean PedsQL™ 4.0 scores for the high risk group to other published data. The high risk group had scores of 44.5 for the Physical Functioning Scale, 70.7 for the Psychosocial Summary Scale, and 61 for the Total Scale. This is placed in clinical perspective by other data showing that scores for children with cancer, in active treatment, are 65, 68, and 67 for the Physical, Psychosocial and Total scales, respectively. [31].

A hypothetical example is presented to illustrate the potential impact of these findings. In a typical health plan, the rate of chronic health conditions will most likely be between 5% [41] and 18% [42], rather than the 31.4% rate we found by selecting our sample, in part, from hospital specialty clinics. We will further conservatively assume that one-fifth (20%) of children with chronic health conditions would fall in the lowest quintile on the PedsQL™ 4.0. If this were so, then between 1% (5% chronic health condition × 20% in the lowest quintile) and 3.6% (18% chronic health condition × 20% in the lowest quintile) of enrollees in a health plan would fall into the high risk group. Thus, in a hypothetical medium to large health plan with 50,000 pediatric enrollees, the high risk group would be comprised of anywhere from 500 (1% of 50,000) to 1800 (3.6% of 50,000) children. Using the costs figures from this sample ($722 pmpm), this hypothetical high risk group represents between $8.6 and $31.2 million in costs over the course of 24 months. This example relies on speculation and is intended as a hypothetical case, for illustrative purposes only.

Taken together, these findings represent an alternative method toward the prospective prediction of healthcare costs in pediatric federally supported managed care populations. While a percentage of these identified costs are inevitable, due to the costs of appropriate care for these chronically ill, poorly functioning children, the possibility exist that a proportion of these healthcare costs are avoidable. Evidence-based disease management has been shown to reduce healthcare costs and increase HRQL in certain chronic conditions such as asthma [43,44]. By identifying at-risk children with low PedsQL™ 4.0 scores, targeted interventions may avert certain future healthcare costs by ameliorating impaired HRQL when first identified.

Certain limitations exist in this study. The first has to do with data not accounted for in this study. We did not have access to pharmacy and mental health costs, nor did we have access to out-of-plan expenditures. For mental health costs, however, recent data has shown that children referred for psychiatric services demonstrate child self-report and parent proxy-report PedsQL™ 4.0 Total Scale Scores comparable to children with chronic physical health conditions [45]. Those data suggest that this methodology may be useful in predicting mental health costs as well. We did not include children under the age of two. Neonatal intensive care, for example, is a large percent of the costs for Medicaid managed care plans [46]. The portion of health plan costs devoted to caring for children under two is not explored here. However, many of these costs cannot be avoided, and preventive efforts for these costs are most appropriately targeted at the prenatal and perinatal periods. Finally, we did not compare the performance of these variables to that of existing administrative risk adjustment methods currently available.

The second limitation has to do with sampling issues and with generalizing these data beyond this study. Our data was not collected at enrollment, nor did we sample from the entire pool of enrollees. Further research is necessary to determine whether these findings hold true for children assessed at health plan enrollment, and to determine the extent to which the results may be influenced by the convenience sample used here. The sample was too small to use cross-validation techniques. Prediction models tend to overfit the development sample, and the predictive validity of these variables should be tested in other, larger samples. Although generalization of these findings to broader populations should be made with caution, the sample here is very diverse with respect to race/ethnicity, and thus likely to be similar to other federally support health plans. We also combined data from two different samples – specialty clinic patients, and health plan members who had been seen in the hospital or outpatient clinics at least three months after the clinical encounter. These two groups could have had unmeasured systematic differences, which could have biased the results.

The third has to do with using a survey to gather these data. The costs of fielding a survey can be quite high, and, if tied to payment, survey responses are subject to "gaming". However, we submit that the potential gains from optimal management of an enrolled population will almost certainly be greater than the costs of survey administration. Moreover, while gaming might occur if health plans were to be compensated based on the HRQL of their enrolled population, the methods used here are suggested as strategies for clinical management, not for rate setting, thus reducing the incentives for gaming. We did not track refusal rates and so do not know what percent of potential participants consented to be in the study. Finally, using a survey means that parents reported on their children's chronic health condition information. Objective measures of chronic health condition would strengthen the validation process. However, in previous PedsQL™ 4.0 clinical research in pediatric patients with cancer, cardiac and rheumatic chronic health conditions, objective medical diagnosis of these chronic diseases demonstrated similar differences between healthy children and children with chronic health conditions as shown in the present findings [28-30].

Further research is necessary. First, a much larger, and randomly selected sample is necessary to confirm these results. Second, split-half validation should be performed so that the coefficients from one group are used to predict the costs in a different group. This could be done with split halves of one large group, or with two similar groups enrolled at different points in time. Given that our regression equation explains 21% of costs, further studies could be done to determine whether other variables might account for additional variance in costs. Further studies could also allow comparison and validation with the results here.

## Conclusion

This is the first study we are aware of to use parent reports of pediatric HRQL and chronic health condition status to prospectively predict healthcare costs in a pediatric sample. These data have implications for healthcare decision makers such as pediatricians, health plan administrators, and policymakers. In a prospective payment system, providers are incentivized to actively manage high-risk patients and to provide care at the appropriate level. The idea behind such a system is that prevention and appropriate care accrues benefits to patients in the form of better health and to providers in the form of lower costs. If, as these data suggest, parent reports of HRQL can be used to predict healthcare costs, one could identify at-risk children proactively and intervene to avoid both illness and costs. In this way, these data can serve simultaneously to improve the health of children and the system that serves them.

## List of Abbreviations

HRQL Health-related Quality of Life

PedsQL™ 4.0 Pediatric Quality of Life Inventory™, Version 4.0

SD Standard Deviation

CCS California Children's Services

URI Upper respiratory infection

PMPM per member per month

## Authors' Contributions

MS, PSK, and JWV conceived of the research. MS and JWV supervised the data collection. MS and DS performed the data analysis. MS drafted the manuscript. JWV, PSK, and DS provided substantive input into the manuscript. All authors read and approved the final manuscript.
